# Absence of Objective Differences between Self-Identified Addicted and Healthy Smartphone Users?

**DOI:** 10.3390/ijerph18073702

**Published:** 2021-04-01

**Authors:** Kristoffer Geyer, Xavier Carbonell, Marta Beranuy, Fran Calvo

**Affiliations:** 1 Department of Psychology, Lancaster University, Lancaster LA1 4YW, UK; 2 FPCEE Blanquerna, Universitat Ramon Llull, 08022 Barcelona, Spain; xaviercs@blanquerna.url.edu; 3 Health Science Department, Universidad Pública de Navarra, 31006 Pamplona, Spain; marta.beranuy@unavarra.es; 4 Departament de Pedagogia, Institut de Recerca Sobre Qualitat de Vida, Universitat de Girona, 17004 Girona, Spain; fran.calvo@udg.edu

**Keywords:** technological addiction, behavioural addiction, smartphone addiction, university students, CERM, self-report measures

## Abstract

Smartphones are used by billions of people worldwide. However, some psychologists have argued that use of this technology is addictive, even though little research utilises objective smartphone usage records to verify this claim. We conducted an exploratory study to identify whether behavioural differences exist between those who self-identify as addicted smartphone users and those who do not. We gathered retrospective smartphone usage data from 131 Android users and asked them about their past use to compare their perception of their usage against their actual usage. We could not identify any reliable differences between the smartphone activity of those self-identified as addicted smartphone users and other users. Furthermore, smartphone scales are generally good at identifying who believes themselves to be addicted, although they do not reflect objective smartphone use. This study questions the use of self-report measures to diagnosis behavioural addictions without relevant psychopathological constructs and emphasises the need for more rigorous study to conceptualise smartphone addiction.

## 1. Introduction

Smartphones are mobile, personal devices that indicate social identity and status with permanent access to the Internet and provide numerous gratifications, such as sociability, entertainment, information finding, time and stress management and social identity maintenance [1,2,3,4,5]. The smartphone has become an essential part of daily life but, in recent years, there has been a surge in literature on smartphone addiction [1,6,7,8,9,10,11], even though no mention has been made of smartphone addiction in either the DSM-5 [12] or in the ICD-11 [13].

In consequence, there is a growing literature about scales that measure this addiction. They have been constructed with high psychometric standards, published in high impact factor journals and used in a wide range of research in the smartphone addiction field. They include, for example, the Mobile Phone Problem Use Scale (MPPUS) [14], the Problematic Mobile Phone Use Questionnaire (PMPUQ) [15], the Cuestionario de Experiencias Relacionadas con el Móvil (CERM) [16], the Smartphone Addiction Scale (SAS) [17], its short version [17], the Cell Phone Addiction Scale (CPAS) [18] and the Smartphone Application-Based Addiction Scale (SABAS) [19].

These scales do not objectively measure smartphone usage; rather, they rely on participants’ self-reports. The smartphone addiction scales have a very low correlation or no reliable correlation with actual smartphone usage [20,21]. Additionally, when more objective measurements of smartphone usage are employed, the smaller the relationship between negative outcomes (anxiety and depression) and smartphone usage [22]. This is a serious problem because of the overreliance on self-report questionnaires to identify smartphone usage. Indeed, all of the studies cited in the previous paragraph about modelling smartphone addiction or linking usage to negative outcomes failed to employ an objective measure of smartphone usage.

This vast body of research uses participants’ self-reports to design questionnaires about smartphone addiction and its correlates, such as depression, anxiety, shyness, stress, self-esteem, loneliness, and so on [23,24]. Factor analyses ensure that the scale is reliable, but validity is far from being established [25]. Remarkably, the symptoms of smartphone addiction have been identified in healthy people (many of them university students) instead of clinical samples (not identified). Additionally, the shortcomings of self-reported assessments are well known [21,25,26,27]. 

An important question about the accuracy of the scales has yet to be answered: What makes them inaccurate? Logically, there are two potential sources of error: (a) the scales are poorly developed (there is evidence to support this claim [26]) and (b) participants struggle to recall their smartphone usage and therefore their interpretations of their smartphone usage is inaccurate.

With this in mind, we aimed to discover whether smartphone users have an accurate impression of their own smartphone usage (by comparing self-report scales, self-identification and objective data) and, for those who self-identify as being addicted to their phone, whether their real usage records reveal a behavioural pattern different from those who do not self-identify as being addicted.

## 2. Materials and Methods

### 2.1. Participants

**Figure 1:**
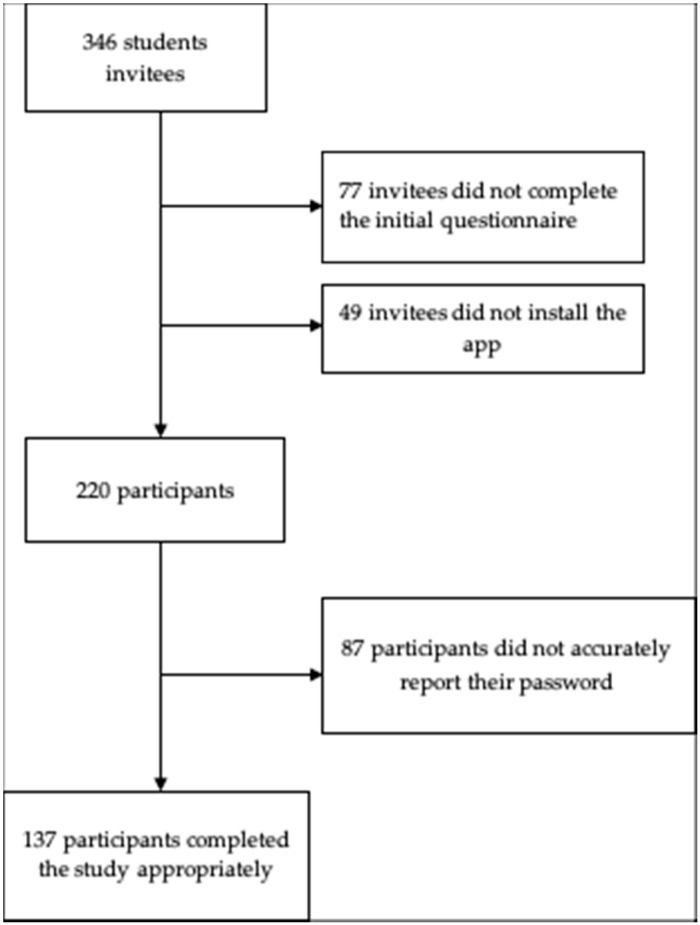
Flow diagram of enrolment and participation.

Three hundred forty-six psychology and social education students from four Spanish universities (Universidad Pública de Navarra, Universitat de Girona, Universitat Ramon Llull and Universidad Internacional de La Rioja) were invited personally by three researchers to participate in the study. To enrol, invitees had to complete a questionnaire about demographics and smartphone usage and install an app. Of the 346 invitees, 77 chose not to begin the enrolment process by completing questionnaires about their demographic data and smartphone usage. Forty-nine invitees who carried out this first enrolment step did not complete enrolment by installing the app. Two hundred twenty invitees completed enrolment by installing the app. Of the 220 enrolled participants, 137 completed the study by sending the data file and the matching password. Six of these participants had not used their smartphone sufficiently over the previous 5 days (they had employed their smartphone on fewer than 3 days during the previous 5 days) to contribute to the study; therefore, the data from 131 participants (age M = 21.9, SD = 7.8) (29 males) were included in this study (see Figure 1 for a flow diagram of enrolment and participation). Our sample size was in line with that of previous studies attempting to compare subjective and objective measures of smartphone use and addiction [20,21,28]. Participants were not rewarded monetarily or academically.

### 2.2. Materials

#### 2.2.1. Sociodemographic and Smartphone Usage Variables

A questionnaire was employed to assess basic sociodemographic characteristics of the participants. The questionnaire further quizzed the participants on their smartphone usage. These questions probed the general frequency with which they used their smartphone for various types of activities (e.g., social media, gambling, etc.). For each activity type, participants were asked if they had associated apps and how heavily they used these apps: rarely, once a day, less than one hour a day or more than one hour a day. These responses were later operationalised to reflect a rough duration, allowing us to assess the accuracy of their self-report.

We also asked participants how they conceptualised their relationship to their smartphone and if they saw their smartphone use to be healthy, problematic or addicted. The participants’ addiction to their smartphone was also assessed using three smartphone addiction scales, as described below.

#### 2.2.2. The “Cuestionario de Experiencias Relacionadas con el Móvil”

[Questionnaire of Mobile phone related experiences] (CERM; [16]). This questionnaire consists of ten items about mobile phone use answered on a four-point Likert scale. Item example: “To what extent do you feel anxious when you do not receive messages or calls?” In the present study, Cronbach’s alpha was 0.72. Cronbach’s alpha in the original study was 0.80.

#### 2.2.3. Fear of Missing Out (FoMO)

The Spanish translation of the FoMO [29] questionnaire was employed [30]. This version consists of ten items to be answered on a 5-point Likert scale from 1 “not at all true of me” to 5 “extremely true of me”. Items typically queried the degree that the participant was concerned they were not included in rewarding social experiences: “I get worried when I find out my friends are having fun without me”; “I fear others have more rewarding experiences than me”. In the present study, Cronbach’s alpha was 0.84.

#### 2.2.4. Smartphone Addiction Scale

Short version (SAS-SV; [17]). This scale, constructed in South Korea, consists of ten items rated from 1 “strongly disagree” to 6 “strongly agree”. The Spanish version of this scale was used [31]. This questionnaire measures whether participants are concerned that they are using their smartphone excessively. Items include: “The people around me tell me that I use my smartphone too much”. The original SAS-SV showed content and concurrent validity and internal consistency (Cronbach’s alpha: 0.91). In the present study, Cronbach’s alpha was 0.85.

#### 2.2.5. The Android App Past Usage

Developed by the first author, utilises the android feature call UsageEvents [32], which maintains a record of all activities that a user carries out for the previous five days. Five days are considered to be sufficient to understand someone’s pattern of smartphone usage [28]. The records indicate, among other things, what apps were installed on the device, when apps were employed, the name of the apps and when the screen was in use. Past Usage queries this record, securely stores the data and packages them for exporting over email. Once all the records were established then the data was packaged in an encrypted pdf and sent to the researcher. The participant’s password was documented in the questionnaire. After the data was collected and relayed to the researcher, the participant was instructed to uninstall the app.

### 2.3. Procedure

The study was approved by the ethics committees of the Universitat Ramon Llull (reference 1819001P) and the Universidad Pública de Navarra (reference PI:003/19). All ethical principles regarding medical research involving human subjects were followed, in accordance with the Declaration of Helsinki. During psychology classes at the universities, students were invited to enrol in the study. On their laptops, they had access to a Qualtrics questionnaire. The questionnaire initially informed them of the nature of the study and then asked to consent to participate. Finally, they installed the app Past Usage on their android device. Instructions on how to do so were provided in the questionnaire. The app required them to generate a password to protect their data and provide permission for the app to access their data. They were notified of the progress of the app in generating the required files. Once the files were encrypted, the participant had to click to send the files to the researchers through email. To do so, they had to provide the password that they had given in the enrolment questionnaire.

### 2.4. Data Analysis

#### 2.4.1. Scoring App Usage

We grouped the apps that the participant used in the last five days, into the previously defined activity types. We included the apps that the participant used for at least 1% of the time they used their screen. In line with subsequent description, this meant an app would have to be used for on average more than 166 s in order to be included in the analysis. This reduced the number of apps to score from over 2000, to roughly 200 separate apps. The apps were ranked by four researchers who looked up the app using the Google Play Store [33] or identified the app using search engines. Then they assessed the app and identified its primary purpose. If the majority of researchers identified an app as belonging to a particular category, it was recorded as belonging as a particular category. If there was a 50/50 split, the app was documented as belonging to both categories.

#### 2.4.2. Smartphone Report Cleaning

Data cleaning followed the following steps: Events that were irrelevant to the actions of the participant were removed from the records (i.e., standby bucket changed, configuration changed, flush to disk, etc.) until three events were all that remained: app moved to foreground, app exited foreground and user interaction. Then we removed any duplication of events. Separate records of an app being in the foreground and a participant interacting with the smartphone for the same event were combined in a single record of active use. Finally, any times that participants left their phone on without actually engaging with it were removed. This was done for non-interactive apps (“bq-Launcher”, “Sistema-Android”, “Android-system”, “app0”, “Nova-Launcher”, “Alarma-de-Lluvia”) being used for a duration of more than 30 min or an app being used without interruption for a duration of an hour and a half.

## 3. Results

### 3.1. Self-Report

Messaging apps, social media and music were reported to be the most frequently used apps. Whereas gambling, adult content and lifestyle were reported to be the most uncommon (see Table 1). The self-reported use of smartphones is presented in Table 2. The participants reported using their phones daily for a mean of 4.8 h a day and checking their phone on average 76 times a day. The mean score on CERM was 17, on SAS-SV 26.5 and 20 on FoMO. The participants characterised their own smartphone usage as healthy (*n* = 54; 40%), problematic (*n* = 70; 51.9%) or addicted (*n* = 11; 8.1%).

### 3.2. Smartphone Usage

**Figure 2:**
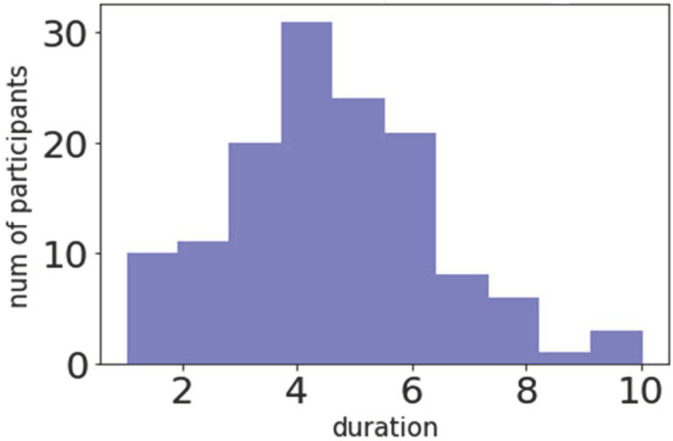
Average smartphone use in the previous five days.

Over the previous five days participants on average used their phone for 5 h and 34 min (see Figure 2) with a standard deviation of 2 h and 8 min. Participants checked their smartphone very frequently (*M* = 138; *SD* = 59). The duration of smartphone usage per activity is displayed in Table 3. Among the 135 participants, over 1708 unique applications were employed by the participants. The number of significant apps (apps used for more than 1% of a person’s screen time) was 382 unique apps across all participants.

### 3.3. Self-Report vs. Smartphone Records

A significant relationship can be consistently found between participants’ objective use of smartphone apps and their self-reported use (see Table 4). However, the strength of the relationship is typically low with the exception of music. If the self-report measurement is operationalised, then we can test the validity of the reported smartphone usage. We operationalised the responses in terms of minutes that the smartphone was used, as follows: I never use it or don’t have it installed = 0; I have it installed but I hardly ever use it = More than 0 and less than 5; Infrequently (once a day or less) = More than 5 and less than 30; Often (several times a day) = More than 30 and less than 60; Very often (more than an hour a day) = More than 60. When employing this operationalisation, we find the following expected likelihood that participants will accurately report their smartphone usage (see Table 5). The activities that were most accurately reported were gambling and betting (96%), adult content (76%), messaging and chatting (63%), social media (57%) and lifestyle (53%).

There was generally a lack of relationship between the smartphone usage scales and the time participants spent on their phone. CERM: *r* = 0.16, *p* = 0.06; SAS-SV: *r* = 0.16, *p* = 0.07; FoMO: *r* = 0.2, *p* = 0.17. There was a stronger relationship between participants self-classification as healthy, problematic or addicted and their actual usage: *r* = 0.25, *p* = 0.003. Similar relationships were found with checking behaviour. CERM: *r* = 0.08, *p* = 0.32; SAS: *r* = 0.11, *p* = 0.19; FoMO: *r* = 0.16, *p* = 0.068. Again, simply asking for an estimate of amount of time checking was more accurate: *r* = 0.19, *p* = 0.025.

**Figure 3:**
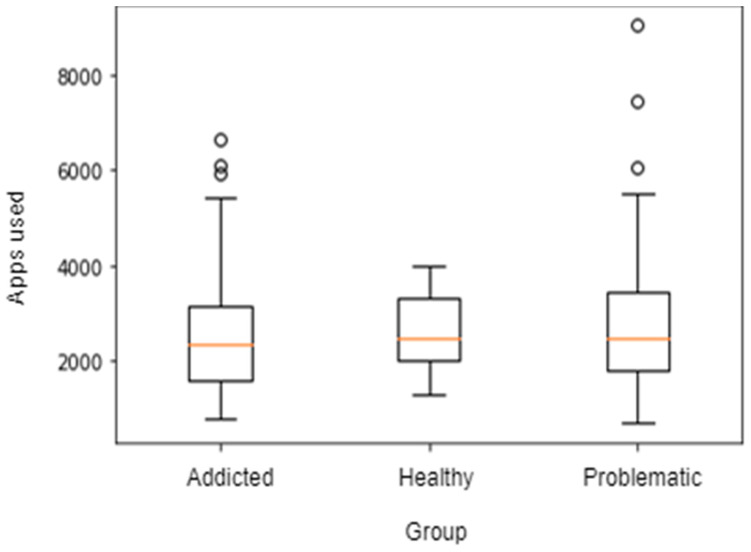
Different overall app usage across types of smartphone users over the previous five days.

**Figure 4:**
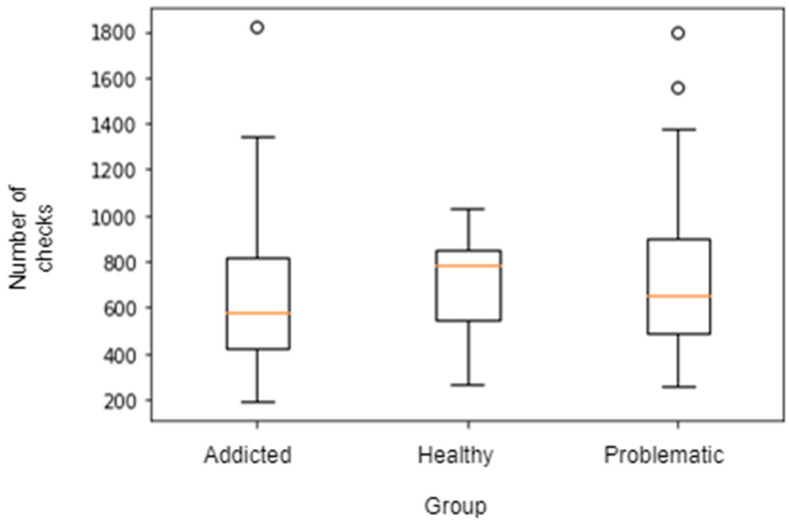
Different overall smartphone checks across types of smartphone users over the previous five days.

**Figure 5:**
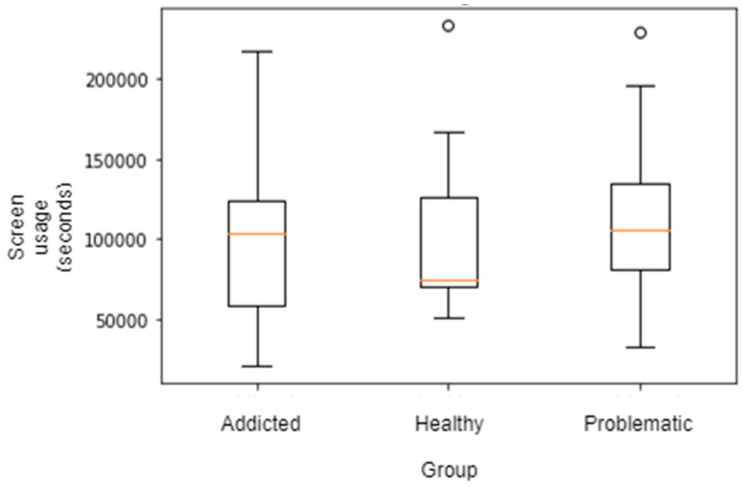
Different overall smartphone usage across types of smartphone users over the previous five days.

However, in this study we identified no reliably significant differences for time spent using a smartphone between those who considered themselves their use healthy vs. problematic (F(1,122) = 2.457, *p* = 0.12), healthy vs. addicted (F(1,63) = 0.228, *p* = 0.635) or problematic vs. addicted (F(1,80) = 0.11, *p* = 0.741). Similarly, the number of times the person picked up their phone was not significantly different between those who self-reported as healthy vs. problematic (F(1,122) = 1.462, *p* = 0.229), healthy vs. addicted (F(1,63) = 0.224, *p* = 0.638) or problematic vs. addicted (F(1,80) = 0.044, *p* = 0.835). Additionally, we found no difference in the number of apps used between people self-reporting as healthy vs. problematic (F(1,122) = 0.535, *p* = 0.466), healthy vs. addicted (F(1,63) = 0.011, *p* = 0.917) or problematic vs. addicted (F(1,80) = 0.104, *p* = 0.748) (see Figure 2). There were some minor behavioural differences. Self-reportedly addicted participants used their smartphone less to call others than self-reportedly healthy participants (F(1,57.918) = 10.796, *p* = 0.0017) and self-reportedly problematic users (F(1,76.71) = 23.06 *p* < 0.00) and used their phones less for music (F(1,33.824) = 9.07, *p* = 0.004) (see Figure 3, Figure 4 and Figure 5).

SAS, FoMO and CERM scores showed significant correlations with participants’ self-classification as healthy, problematic or addicted smartphone user. For healthy and problematic sub-samples, the distribution was significantly different, as measured by the Levene test. For FoMO, test scores were significantly different for those who self-declared as healthy vs. problematic (F(1,119.296) = 17.603, *p* < 0.001)) and for healthy vs. addicted (F(1,16.422) = 16.717, *p* < 0.001)) but not for problematic vs. addicted (F(1,16.808) = 1.513, *p* = 0.236)). CERM test scores were significantly different for those who self-declared as healthy vs. problematic (F(1,12.732) = 36.171, *p* < 0.001) and for healthy vs. addicted (F(1,16.422) = 16.717, *p* < 0.001)) but not for problematic vs. addicted (F(1,80) = 20.24, *p* < 0.001)). SAS tests scores were significantly different for those who self-declared as healthy vs. problematic (F(1,109.118) = 17.851, *p* < 0.001) and for healthy vs. addicted (F(1,19.38) = 70.991, *p* < 0.001)) and for problematic vs. addicted (F(1,80) = 21.29, *p* < 0.001)).

The results of our attempt to use the smartphone addiction questionnaires to detect the profiles of smartphone usage were underwhelming. The questionnaires could not predict smartphone usage, number of checks or number of app changes (see Table 6). Of the 22 smartphone activity types tested for, the scales could identify usage of social media (CERM (ρ (134) = 0.188, *p* = 0.029); SAS (ρ (134) = 0.20, *p* = 0.018); FoMO (ρ (134) = 0.21, *p* = 0.014)). Additionally, some could predict use of organisational apps (CERM (ρ (134) = 0.20, *p* = 0.023); FoMO (ρ (134) = 0.18, *p* = 0.039)). SAS could also predict use of entertainment apps (ρ (134) = −0.19, *p* = 0.032).

## 4. Discussion

This study reveals that those who self-identify as being addicted or score high for addiction on self-report questionnaires do not present a significantly different smartphone usage pattern from those who do not self-identify as being addicted. They do not use or pick up their phone reliably more than other participants and the number of apps employed is not different. We only identified differences of little consequence: the self-declared addicted spent less time on calls and listening to music. No distinctive behaviour was found for those with an addicted or problematic relationship relative to those with a healthy relationship (according to self-identification of self-report questionnaires) [20,21,34]. We are therefore sceptical that individuals can accurately self-diagnose their relationship with their smartphone [26].

Another important finding is that participants who considered themselves healthy had significantly different scores on the CERM, FoMO and SAS-SV from participants who reported problematic use or addiction. But only SAS-SV was able to identify the difference between those who considered themselves problematic vs. addicted, confirming that SAS-SV is a reliable instrument [31,35,36]. These results would suggest that the scales worked effectively to capture the self-concept of the individual. However, because self-concept is not based on real use, the value of the scales as tools to assess objective reality (which they are frequently used for) is limited.

This study is important because its theoretical framework is based on the use of objective measures of smartphone use instead of the usual psychometric scales [21,34,37]. We have been able to replicate past findings showing that objective measures of smartphone usage only weakly correlate with self-report measures [20] and that smartphone usage records do not correlate with questionnaires aimed at assessing smartphone usage [20,21]. Interestingly, our findings contradict those of Lin et al. [34], in which the daily use count and the trend of this frequency were associated with smartphone addiction.

### 4.1. Activities on the Phone

University students reported that the most frequent activities for which they used their smartphones were messaging apps, social media and listening to music. Moreover, the Past Usage app identified messaging apps, social media, news, gaming and organisation as frequent activities. Interestingly, the distribution of smartphone activities are similar to those found in similar cohorts [38,39], with the exception of a lower incidence of shopping behaviours. These results make evident the overlap between the phone and the Internet [38,40]. That is, participants used the phone to access the Internet and they reported that they invested more time in this activity than talking, and, moreover, this perception is confirmed by objective data.

Gaming is a fairly frequent activity among students [41], especially among men, but women have joined this activity mainly because the smartphone is the logic platform for casual games (designed for accessibility, simplicity and the speed with which rules can be grasped), such as ‘Candy Crush’ or ‘Pou’ and unlike games such as ‘League of Legends’ and ‘World of Warcraft’, whose most suitable game platform is the personal computer. However, contrary to expectations [42,43], gambling has not been recognised by users or by Past Usage as a frequent activity; once again, the high proportion of women in the sample could be the explanatory factor for this phenomenon. Significant gender differences were found between all uses except for online purchases, viewing of TV series, movies or videos, and organisational tasks.

### 4.2. Smartphone Usage

The self-reported time of smartphone use (4.8 h per day), is approximately 10% less than the real time, and the number of real checks (138 real vs. 76 self-reported) is surprisingly higher than that reported in other studies [20,21]. The explanation might lie in the fact that many checks are undertaken unconsciously. When using the objective measure, messaging, emails and social media were the predominant activity in student life. Information seeking and videogames also emerged as frequent activities, followed by multimedia and music, while academic activities were less frequent.

### 4.3. Self-Perception and Objective Measures of Addiction

We have used several measures to evaluate the behavioural relationship of the participants with their smartphones. The CERM was designed in 2009 [16] in the period of transition from mobile phone to smartphone. Despite this shift, it remains valid because it focuses on perception of the experiences caused by the phone without distinguishing its technological potentialities. The SAS was designed in Taiwan for use with adolescents, and both the long [5] and short [17] versions have been used specifically to assess possible addiction. For its part, the FoMO measures fears and worries about missing out on rewarding experiences with others (e.g., going out with friends [29]).

CERM scores in college students increased between 2009 and 2020 [3,16,38,40,44] with a tendency to stabilise in the last five years at around 17 points. This score can be considered in the low-mid range of the scale, although the authors have never established a cut-off point that indicates an addictive disorder or other psychological problem. The mean scores of our sample in the SAS are in line with previous research [17,31], at around 27 points and below the 30 points that the authors suggest as a cut-off [17] and the 39.8 found in a sample of Brazilian adolescents [35]. Regarding the scores on the FoMO, the results of our sample are practically identical to those obtained in the Spanish adaptation of Oberst [30] (FoMO: 20.4, SD: 6.68) and slightly lower than those of other studies that have used this scale [45] (FoMO: 22.04; SD: 7.51); [46] (FoMO: 21.27; SD: 7.24)

Generally, these results indicate that while the smartphone scales can reliably identify those who consider themselves to be addicted or not, they are not useful when identifying actual use. This is because those who are identified as addicted (either by self-classification or via a smartphone addiction scale) do not differ in regard to usage, checks or number of app changes when compared to those who are not identified as addicted. Only when we look at specific social media usage can the scales identify with any accuracy a difference in smartphone usage across these types of user. Given the difficulty of finding objective differences across supposed user types, it is essential to conduct further research to describe frequent smartphone activities, for example social media or gaming, to understand why some people consider that their use of the phone is unhealthy [39,40].

This inconsistency between self-report measures and objective measures can be applied to other behavioural addictions. There is growing evidence that the scales used to measure behavioural addictions based on self-report lack validity and do not reflect psychopathological disorders, despite the fact that they are developed using the current standards of validity and reliability [21,47]. Another factor that may explain this inconsistency is that social desirability could bias the participants’ responses [48,49]. Our critical appraisal suggests that we should re-examine studies that purport to measure addiction to things such as offline friends [47], the Star Wars universe [50], Harry Potter books [51] or tango dancing [52].

This study is not without limitations. Some of the students that we invited to participate were very reluctant to allow the app to access data on their phone, and the extensive security procedure resulted in a large number of invitees failing to complete the enrolment process and send their smartphone data. Therefore, the sample was smaller than we would have liked (although it is in line with that of similar studies [20,21,28,34]), and it may disproportionally include those who are laxer about their privacy. However, it is unlikely that laxity vs. strictness about privacy would affect the study outcome variables. Additionally, our sample of participants who considered themselves addicted was very small and women were overrepresented, therefore limiting the analysis. Nonetheless, sample size was adequate given the vast amount of data collected for each participant. The findings of this study should be replicated with a significantly larger participant pool.

## 5. Conclusions

In this exploratory study, we asked whether participants could accurately classify their relationship with their smartphone, based on their perception of their smartphone usage in comparison to their actual usage. Participants’ conceptualisation of their relationship with their smartphone was unrelated to their actual behaviour. Regardless, the self-report scales distinguished group differences in the conceptualisation of a healthy, problematic or addicted user. The scales thus seem accurate at identifying individuals’ subjective assessment of themselves, but they do not reflect objective use and are therefore not useful as an instrument for diagnosing smartphone addiction. More rigorous study is required to conceptualise smartphone addiction.

## Figures and Tables

**Table 1:** Activities on the Smartphone.

Activities	Response (Explained below)				
	1	2	3	4	5
Phone calls and videoconferences	7	31	71	23	3
Messaging and chatting (e.g., WhatsApp, Telegram)	1	0	3	37	94
Social media (e.g., Facebook, Twitter, Instagram, Tinder, LinkedIn, YouTube)	2	2	8	45	78
General information (e.g., news, sports, weather, politics)	9	23	56	41	6
Online shopping (e.g., clothes, food, Amazon)	58	63	7	6	1
Games and video games	62	35	14	13	11
Gambling and betting (e.g., poker, bingo, casino)	129	4	0	1	1
Multimedia (e.g., Netflix, TV series, films)	20	17	42	33	23
Music (e.g., Spotify)	12	7	22	44	50
Administrative tasks (e.g., banks, payments, tickets)	29	50	44	11	1
Adult content: pornography, eroticism	103	11	19	2	0
Education and academic activities (e.g., books, library, dictionary, information search)	23	25	49	31	7
Working activities (e.g., word processing)	35	34	43	16	7
Lifestyle (e.g., home, cars, beauty)	74	25	24	11	1
Maps, GPS and public transport	6	56	56	15	2
Health (e-g., diseases, nutrition, pharmacy)	64	34	26	11	0
Organisational tasks (calendar)	12	40	49	29	5
Other apps	46	34	45	9	1

List of responses: 1. Nunca o no la tengo instalada (I never use it or don’t have it installed). 2. La tengo instalada pero casi nunca la uso (I have it installed but I hardly ever use it). 3. Con poca frecuencia (1 vez al día o menos) (Infrequently (Once a day or less)). 4. Con frecuencia (varias veces al día) (Often (several times a day)). 5. Con mucha frecuencia (más de una hora al día) (Very often (more than an hour a day)).

**Table 2:** Self-Reported Usage of Smartphones.

	Mean (SD)	Median	Range
How many hours do you use your phone daily?	4.8 (2.6)	5	1–21
How many times do you check your phone daily?	76 (68)	50	10–99
CERM	17 (3.7)	17	11–36
SAS-SV	26.5 (9.6)	25	10–56
FoMO	20 (5.75)	20	10–46

**Table 3:** Activities on the Smartphone.

Activities	Duration (Seconds)	
	*M*	*SD*
Phone calls and videoconferences	1307	1844
Messaging and chatting (e.g., WhatsApp, Telegram)	34,423	22,834
Social media (e.g., Facebook, Twitter, Instagram, Tinder, LinkedIn, YouTube)	63,189	30,122
General information (e.g., news, sports, weather, politics)	7644	8416
Online shopping (e.g., clothes, food, Amazon)	242	1102
Games and video games	4617	10,032
Gambling and betting (e.g., poker, bingo, casino)	0	0
Multimedia (e.g., Netflix, TV series, films)	2101	5633
Music (e.g., Spotify)	2683	4765
Administrative tasks (e.g., banks, payments, tickets)	59	180
Adult content: pornography, eroticism	22	256
Education and academic activities (e.g., books, library, dictionary, information search)	680	1812
Working activities (e.g., word processing)	811	1474
Lifestyle (e.g., home, cars, beauty)	96	494
Maps, GPS and public transport	567	889
Health (e.g., diseases, nutrition, pharmacy)	1063	5182
Organisational tasks (calendar)	2537	8708

**Table 4:** Response to Questionnaires about App Usage by Activity Type.

Activity Type	Male					Female				
	1	2	3	4	5	1	2	3	4	5
1	0	8	16	5	0	6	23	55	18	3
2	0	0	1	11	17	0	0	2	26	77
3	1	0	1	12	15	0	2	7	33	63
4	3	4	10	10	2	5	19	46	31	4
5	14	13	1	0	1	43	50	6	6	0
6	8	6	2	7	6	53	29	12	6	5
7	25	2	0	1	1	103	2	0	0	0
8	4	5	5	4	11	15	12	37	29	12
9	0	1	4	10	14	11	6	18	34	36
10	7	8	14	0	0	21	42	30	11	1
11	12	3	12	2	0	90	8	7	0	0
12	6	8	9	6	0	16	17	40	25	7
13	5	9	11	2	2	29	25	32	14	5
14	19	5	5	0	0	54	20	19	11	1
15	3	13	12	1	0	2	43	44	14	2
16	22	4	3	0	0	41	30	23	11	0
17	5	11	7	6	0	6	29	42	23	5
18	9	6	13	1	0	36	28	32	8	1

*Key.* The activity type numbers in the first column correspond with the following activity types: 1. Phone calls and video conferences; 2. Messaging and chatting (e.g., WhatsApp, Telegram); 3. Social media (e.g., Facebook, Twitter, Instagram, Tinder, LinkedIn, YouTube); 4. General information (e.g., news, sports, weather, politics); 5. Online shopping (e.g., clothes, food, Amazon); 6. Games and video games; 7. Gambling and betting (e.g., poker, bingo, casino); 8. Multimedia (e.g., Netflix, TV series, films); 9. Music (e.g., Spotify); 10. Administrative tasks (e.g., banks, payments, tickets); 11. Adult content: pornography, eroticism; 12. Education and academic activities (e.g., books, library, dictionary, information search); 13. Working activities (e.g., word processing); 14. Lifestyle (e.g., home, cars, beauty); 15. Maps, GPS and public transport; 16. Health (e.g., diseases, nutrition, pharmacy); 17. Organisational tasks (calendar); 18. Other apps.

**Table 5:** Relationship between Self-Reported Activities on the Smartphone and Objective Behaviour (Past Usage).

Activities	*r*	*P*
Phone calls and video conferences	0.20	*0.022*
Messaging and chatting (e.g., WhatsApp, Telegram)	0.34	0.000
Social media (e.g., Facebook, Twitter, Instagram, Tinder, LinkedIn, YouTube)	0.26	0.002
General information (e.g., news, sports, weather, politics)	0.21	0.012
Online shopping (e.g., clothes, food, Amazon)	0.17	0.044
Games and video games	0.63	0.000
Gambling and betting (e.g., poker, bingo, casino)	NA	NA
Multimedia (e.g., Netflix, TV series, films)	0.17	0.044
Music (e.g., Spotify)	0.63	0.000
Administrative tasks (e.g., banks, payments, tickets)	0.36	0.000
Adult content: pornography, eroticism	−0.048	0.583
Education and academic activities (e.g., books, library, dictionary, information search)	0.00	0.99
Working activities (e.g., word processing)	0.21	0.016
Lifestyle (e.g., home, cars, beauty)	0.06	0.53
Maps, GPS and public transport	0.37	0.000
Health (e.g., diseases, nutrition, pharmacy)	0.23	0.008
Organisational tasks (calendar)	0.23	0.009
Other apps	0.03	0.71

**Table 6:** Accuracy of Self-Reported Activities on the Smartphone and Objective Behaviour (Past Usage).

Activities	Accuracy (%)
Phone calls and video conferences	28
Messaging and chatting (e.g., WhatsApp, Telegram)	63
Social media (e.g., Facebook, Twitter, Instagram, Tinder, LinkedIn, YouTube)	57
General information (e.g., news, sports, weather, politics)	34
Online shopping (e.g., clothes, food, Amazon)	44
Games and video games	45
Gambling and betting (e.g., poker, bingo, casino)	96
Multimedia (e.g., Netflix, TV series, films)	16
Music (e.g., Spotify)	17
Administrative tasks (e.g., banks, payments, tickets)	33
Adult content: pornography, eroticism	76
Education and academic activities (e.g., books, library, dictionary, information search)	16
Working activities (e.g., word processing)	30
Lifestyle (e.g., home, cars, beauty)	53
Maps, GPS and public transport	33
Health (e.g., diseases, nutrition, pharmacy)	46
Organisational tasks (calendar)	28
Other apps	26

## Data Availability

Not applicable.
